# Infection of hepatitis B virus in extrahepatic endothelial tissues mediated by endothelial progenitor cells

**DOI:** 10.1186/1743-422X-4-36

**Published:** 2007-04-02

**Authors:** Qifei Rong, Jun Huang, Enben Su, Jun Li, Jianyong Li, Lili Zhang, Kejiang Cao

**Affiliations:** 1 Department of Cardiology, First Affiliated Hospital of Nanjing Medical University, Nanjing 210029, People's Republic of China; 2 Department of Infectious Disease, First Affiliated Hospital of Nanjing Medical University, Nanjing 210029, People's Republic of China; 3 Department of Hematology, First Affiliated Hospital of Nanjing Medical University, Nanjing 210029, People's Republic of China

## Abstract

**Background:**

Hepatitis B virus (HBV) replication has been reported to be involved in many extrahepatic viral disorders; however, the mechanism by which HBV is trans-infected into extrahepatic tissues such as HBV associated myocarditis remains largely unknown.

**Results:**

In this study, we showed that human cord blood endothelial progenitor cells (EPCs), but not human umbilical vein endothelial cells (HUVECs) could be effectively infected by uptake of HBV in vitro. Exposure of EPCs with HBV resulted in HBV DNA and viral particles were detected in EPCs at day 3 after HBV challenge, which were peaked around day 7 and declined in 3 weeks. Consistently, HBV envelope surface and core antigens were first detected in EPCs at day 3 after virus challenge and were retained to be detectable for 3 weeks. In contrast, HBV covalently closed circular DNA was not detected in EPCs at any time after virus challenge. Intravenous transplantation of HBV-treated EPCs into myocardial infarction and acute renal ischemia mouse model resulted in incorporation of HBV into injured heart, lung, and renal capillary endothelial tissues.

**Conclusion:**

These results strongly support that EPCs serve as virus carrier mediating HBV trans-infection into the injured endothelial tissues. The findings might provide a novel mechanism for HBV-associated myocarditis and other HBV-related extrahepatic diseases as well.

## Background

As many as 20% of patients with hepatitis B virus (HBV) infection experience a spectrum of extrahepatic disorders that includes dermatologic disease, polyarthralgias and arthritis, glomerulonephritis, polymyositis, aplastic anemia, neuropathy, vasculitis and myocarditis [1-3]. Recent studies revealed that the virus has extensive reservoirs of extrahepatic replication [4]. HBV proteins and nucleic acids have been found in a number of non-hepatic tissues including lymph nodes, spleen, bone marrow, kidney, colon, stomach, periadrenal ganglia, skin, thyroid, pancreas, testis, ovaries, brain, heart and lung tissue [5-8]. It is likely that many different cell types such as endothelial cells, epithelial cells, neurons, macrophages, bone marrow cells, peripheral blood mononuclear cells and polymorph nuclear leukocytes are permissive for HBV replication in humans [4,7,9-11]. Recently, HBV replication was found in damaged endothelial tissues of patients with extrahepatic disease [12], which indicates that endothelial tissues may be one of the tropism tissues infected by HBV in extrahepatic disease. However, in contrast, several other studies have demonstrated that the HBV is not replicated in peripheral blood mononuclear cells (PBMCs) [13], endothelial cells [14], and lymphatic tissues [15]. Therefore, whether HBV could be replicated in extrahepatic tissues remains controversial.

Endothelial progenitor cells (EPCs) are primitive cells made in the bone marrow that can enter the bloodstream and go to areas of blood vessel injury to help repair the damage. It should be pointed out that EPCs not only exist in adult bone marrow, but also exist in blood circulation and peripheral cord blood [16,17]. Emerging evidence suggests that EPCs are able to differentiate into mature endothelial cells, contribute to neovascularization and reendothelialization during both embryonic and postnatal physiological processes [16-21]. Despite bone marrow-derived cells including hematopoetic stem cells and peripheral blood mononuclear cells were recently shown to support HBV replication, the subset of these cells such as EPCs has not been explored. It is possible that EPCs are also permissive for HBV uptake or replication. Therefore, in this study we tested whether EPCs from human umbilical cord blood can be infected with HBV in vitro. The data provided in this study show for the first time that EPCs can be effectively infected by uptake of HBV in vitro. Using myocardial infarction (MI) mouse model induced by ligation of coronary artery and acute renal ischemia mouse model induced by unilateral renal artery clamping, we could show that transplantation of EPCs with HBV in mice leads to HBV tans-infection into injured extrahepatic endothelial tissues in heart, lung, and kidney through the processes of EPCs recruitment. Taken together, our results suggest that the harboring of HBV in EPCs could serve as one of extrahepatic infective sources, which might point to a novel role of EPCs in mediating HBV associated myocarditis and other HBV-related extrahepatic diseases as well.

## Results

### Characterization of EPCs

**Figure 1:**
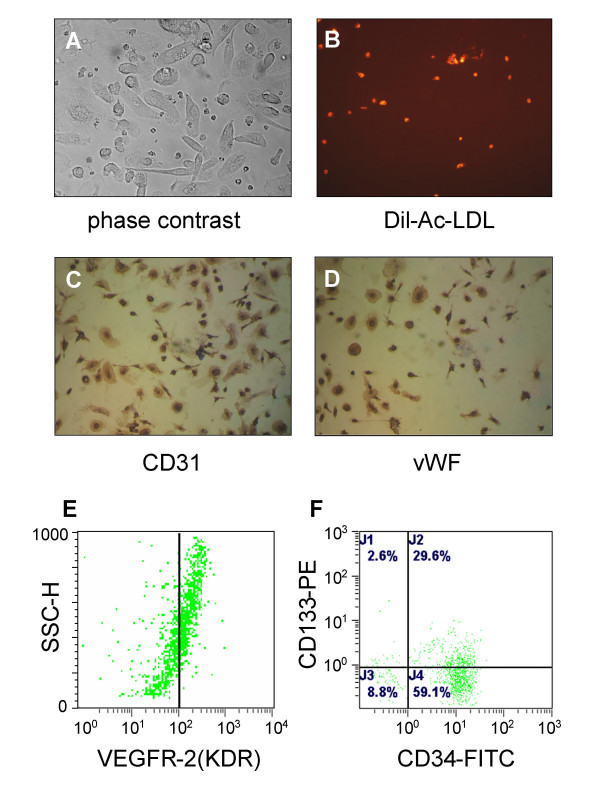
**Characterization of EPC**. Isolated EPCs were visualized by phase-contrast microscope (A 400 ×), and DiI-Ac-LDL uptake was analyzed by fluorescent microscope (B 100 ×). The cells were further analyzed by immunostaining with anti-CD31(C 200 ×) and vWF antibodies(D 200 ×), or by flow cytometry with anti-KDR (E) and CD34/CD133 antibodies (F).

**Figure 2:**
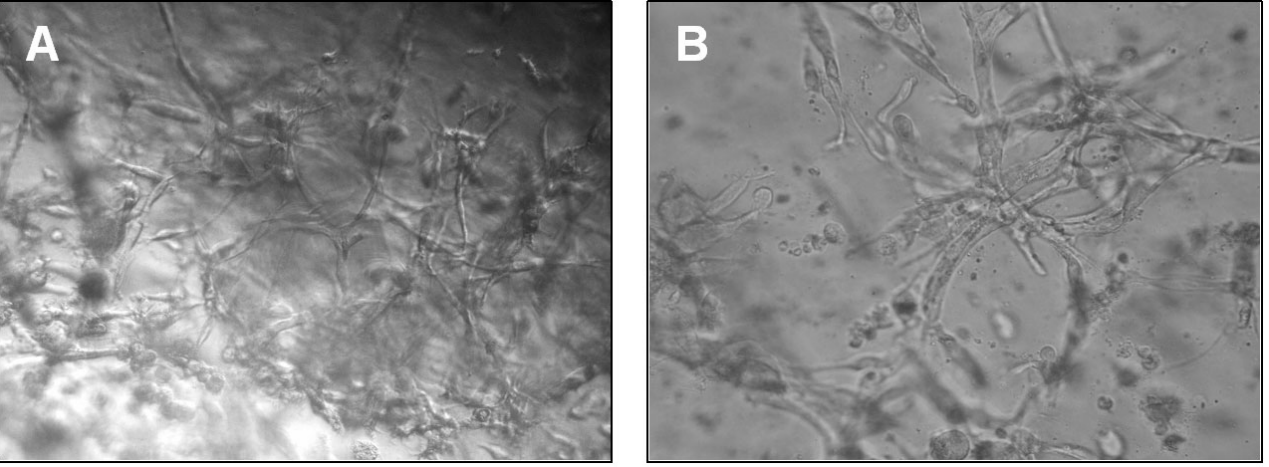
**Effect of HBV infection on tubules formation of EPCs**. Matrigel was polymerized on 96-well plates, and 1 × 10^4 ^of EPCs were plated in Medium 199 containing either 10% normal control serum (mock-treated, A) or10% HBV-positive serum (HBV-treated, B), and incubated at 37°C for 72 hrs. The tubules formation was visualized by an inverted microscope. Magnification for panel A, × 400; for panel B, × 600.

EPCs showed a spindle-shaped, endothelial cell-like morphology after 7 days culture in Medium-199 (Fig. 1A). EPCs were capable of uptaking DiI-ac-LDL (Fig. 1B). Immunohistochemistry showed that the cells were positive for endothelial cell surface markers such as vWF and CD31 expression (Fig. 1C and 1D). Furthermore, flow cytometric analysis showed that 81.7 ± 12.2% of the cells were positive for CD34, 37.5 ± 10.6% were positive for CD133 and 48.2 ± 8.1% positive for KDR expression (Fig. 1E and 1F). In addition, EPCs were capable of forming capillary tubule-like structures on Matrigel (Fig. 2A). In contrast to EPCs, HUVECs grew close to one another in parallel arrays and were positive for vWF and CD31 expression, and contained cytoplasmic inclusions (Weibel-Palade bodies) characteristic of in situ endothelial cells (data not shown).

### HBV infection had no effect on angiogenic capacity of EPC

The effect of HBV on endothelial network formation of EPCs was further determined in vitro tubule formation assay. HBV-treated EPCs were observed for initiation and maintenance of capillary tubule-like structures over 3 days (Fig. 2). The capillary-like structures were similar between mock-treated and Virus-treated EPCs (19.3 ± 1.78 for mock-treated vs.19.5 ± 2.31 for virus-treat, n = 20, p > 0.05). The results suggested that HBV infection may not affect EPCs potential function on tubulogenesis and angiogenesis.

### EPC is a target for HBV infection

**Figure 3:**
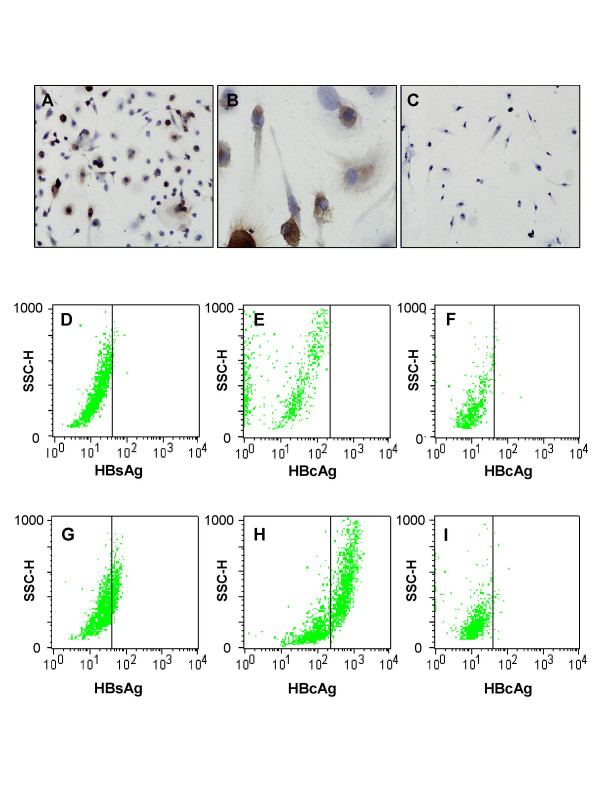
**Expression of HBsAg and HBcAg antigens in EPCs**. EPCs (A, B, D, E, G, and H) and HUVECs (C, F, and I) were co-cultured with HBV (A-C, and G-I) for 4 days, washed and maintained for another 3 days or without virus (D-F) for 7 days. The cells were either immunostained with anti-HBcAg antibody (A and C, × 200; and B, × 600), or analyzed by flow cytometry with anti-HBsAg (D and G) and anti-HBcAg antibodies (E, F, H and I).

To determine whether EPC could be a target for HBV infection, cells were immediately exposed to either HBV positive serum (virus-treated) or non-virus control serum (mock-treated) after initial seeding. HBcAg was first detectable at day 3 after infection with HBV (Fig. 3A), and retained to be detectable for 3 weeks (data not shown). HBcAg was predominantly found in the cytoplasm of infected cells (Fig. 3B). Flow cytometric analysis showed that 33.5 ± 9.1% (n = 5) of EPCs were positive for HBsAg (Fig. 3G), and 40 ± 17.3% (n = 5) of EPCs were positive for HBcAg (Fig. 3H), respectively. As expected, no HBsAg and HBcAg expression found in mock-treated EPCs (Fig. 3D and 3E) and HUVECs (Fig. 3F). It is worth noting that there was no HBcAg expression observed in HUVECs at any time points after exposure to HBV by either immunohistochemistry (Fig. 3C) or by flow cytometric analysis (Fig. 3I).

**Figure 4:**
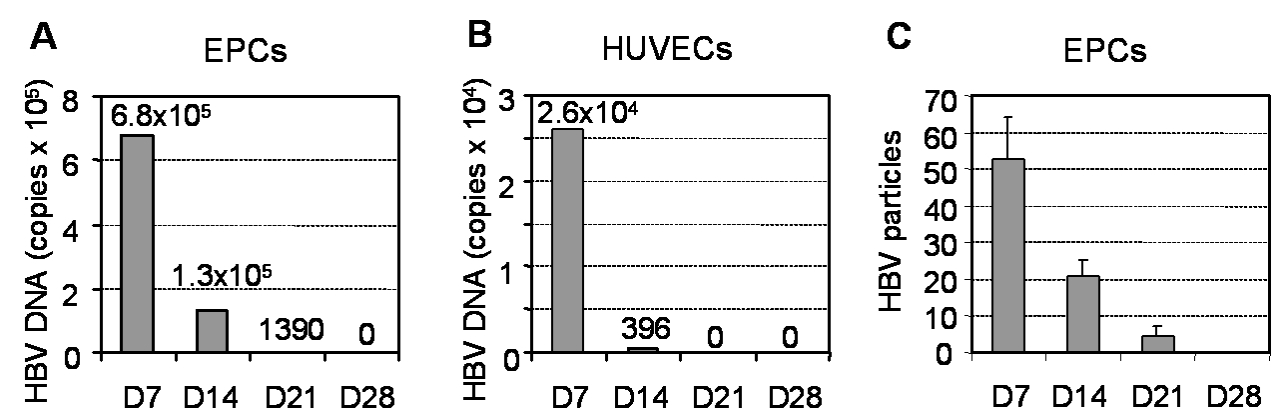
**Time dependent expression of HBV in EPCs**. Detection of HBV DNA and HBV particles in EPCs (A and C) and HUVECs (B) by qRT-PCR analysis (A and B) and by transmission electron microscopy (C) at various times as indicated. The viral particles were calculated under transmission electron microscopy (× 20,000) from 5 different fields. D = Day.

The infection of EPC with HBV was also evidenced by virus DNA detection. Seven days after infection of cells with virus positive serum (10^8 ^copies of HBV DNA/10^6 ^cells), we could detected about 6.8 × 10^5 ^copies of HBV DNA/10^6 ^cells in the primary EPC culture (Fig. 4A). However, further cultivation of cells resulted in a rapid decrease in HBV DNA in EPCs, which was completely disappeared in 4 weeks after virus challenge (Fig. 4A). In contrast to EPC, there was only lower copies of HBV DNA (2.6 × 10^3 ^copies/10^6 ^cells) could be detected in the primary HUVECs culture, and no any trace of virus DNA was detected in HUVECs after first passage (Fig. 4B). These results support that EPC rather than HUVEC is more susceptible to HBV uptake and infection. Significantly, no HBV cccDNA could be detected in EPC at any time after HBV infection (data not shown).

**Figure 5:**
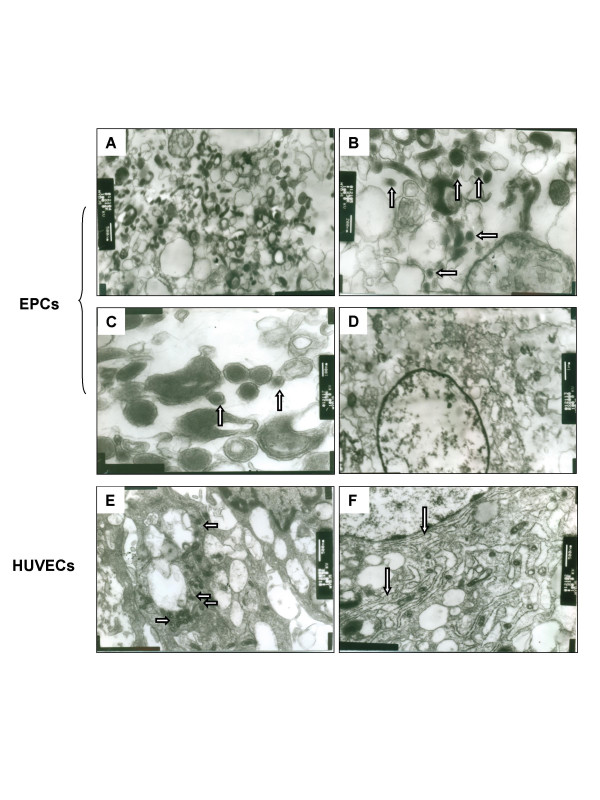
**Detection of HBV viral particles in EPCs**. HBV-treated (A, B, and C) or mock-treated EPCs (D) or HBV-treated HUVECs (E and F) were cultured in Medium 199 for 7 days and the cells were analyzed by transmission electron microscope. Arrows indicate the virus particles with a diameter of 80 nm in B and C, vesicle in E, and rough endoplasmic reticulum (RER) in F. Bars are 500 nm in A, E, and F; 200 nm in B; 100 nm in C; and 1 μm in D.

Using transmission electron microscopic analysis, HBV-like particles could be detected in the virus-treated EPCs. As shown in Fig. 5A–5C, HBV particles were roughly spherical with a variable diameter of 50 to 200 nm. Many of them with a diameter of 80 nm, and some of them showed 80 nm width and 200 nm length like filaments of HBsAg. These particles were localized exclusively in cytoplasm but not in rough endoplasmic reticulum (RER) and nucleus. The virus particles were peaked at day 4–7 after infection, and were reduced to half at day 14. Only few virus particles were observed at day 21, and they were completely disappeared after day 28 (Fig. 4C). No virus particle could be detected in mock treated-EPCs (Fig. 5D) and in virus-treated HUVECs (Fig. 5E–5F), indicating that infection of HBV is a predominant phenomenon of EPCs. It is worth noting that there were more vesicles and RERs in the cytoplasm of HUVECs (Fig. 5E and 5F) when compared to EPCs (Fig. 5D).

### HBV is trans-infected into heart, lung, and kidney through the process of EPC recruitment

**Figure 6:**
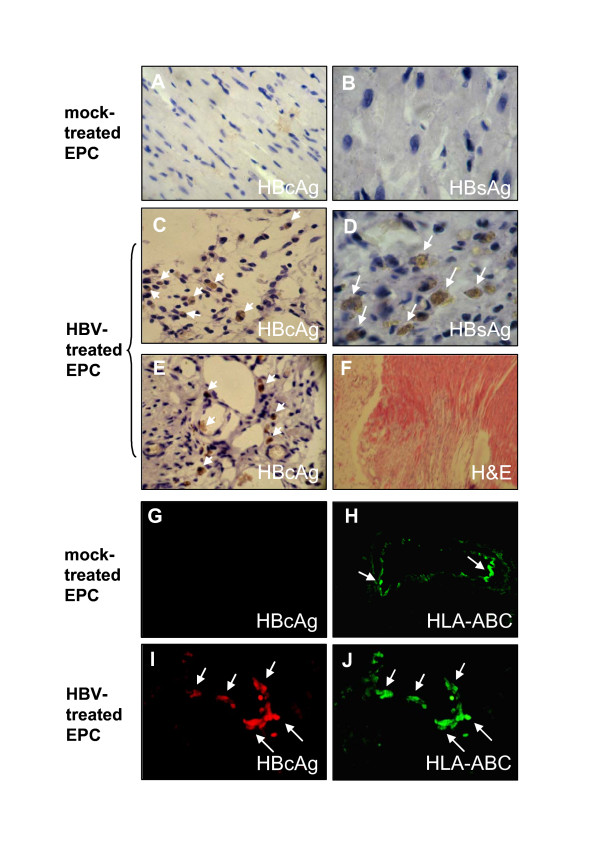
**Detection of HBV antigens in mouse heart tissues**. Two weeks after cell transplantation, hearts were harvested, immunostained with anti-HBcAg (A, C, and E) and anti-HBsAg (B and D). Myocardial interstitium (A-D) and intra-myocardial capillaries (E) in the peri-infarct area were shown. Hematoxylin and eosin (H&E) staining of heart tissue after 2 weeks MI-induction revealed a clear infract area (F). Magnification for panels B and D, × 800; for panels A, C and E, × 400; and for panel F, × 40. In addition, hearts were double immunostained with HBcAg (G and I) and human HLA-ABC (H and J) 3 weeks after cell transplantation as described in "Materials and Methods". Intra-myocardial capillaries in the peri-infarct area were shown. Magnification × 400. Mice transplantated either with mock-treated EPCs (A, B, G, and H) or with HBV-treated EPCs (C-E, I and J) were indicated. Results are representative of at least three independent experiments.

Finally, we examined whether HBV can be transmitted to animal tissues by in vivo transplantation of EPCs into MI mouse model. Our data showed HBV-treated EPCs had a higher capacity to induce neovascularization. Both HBsAg and HBcAg were observed in the interstitial tissues (Fig. 6C and 6D) and capillaries (Fig. 6E), in peri-infarct areas after cell transplantation for 2 weeks. Neither HBcAg nor HBsAg was found in mouse heart tissues transplanted with mock-treated EPCs (Fig. 6A and 6B).

To further address whether the virus-positive cells in the tissues are derived from human EPCs, a human HLA-ABC antibody was used to identify the human major histocompatibility complex (MHC). As expected, HBcAg was not found in mouse heart tissues transplanted with mock-treated EPCs (Fig. 6G). However, in the HBV-treated EPCs transplantation, HBcAg antigen was exclusively co-expressed with human MHC positive cells (stained by HLA-ABC) in the intramyocardial capillaries in the peri-infarct area (Fig. 6I and 6J). No HBV DNA was found in blood when mice were transplanted with HBV-treated EPCs, and no HBV antigens were found in heart tissues in both MI and sham-operated mice received only virus transplantation (data not shown). These results clear indicated that the HBV positive cells found in the injured capillary endothelial tissues were differentiated from the transplanted human EPCs.

**Figure 7:**
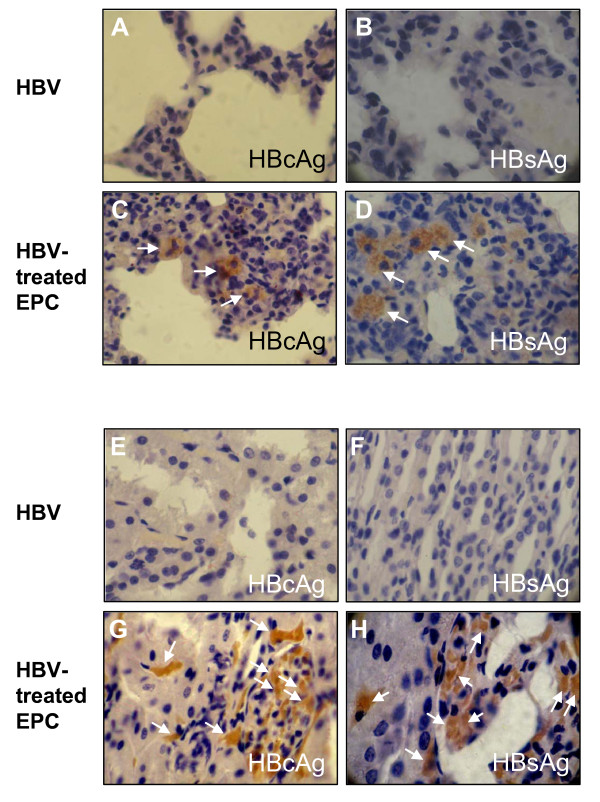
**Detection of HBV antigens in mouse lung and kidney tissues**. Lung interstitium (A-D) in MI mice and renal glomerular vascular endothelium (E-H) in MI mice with acute renal ischemia were immunostained with anti-HBcAg (A, C, E and G) and anti-HBsAg (B, D, F and H) after 2 weeks of cell transplantation. Magnification × 800. Mice transplantated either with HBV alone (A, B, E and F) or with HBV-treated EPCs (C, D, G and H) were indicated. Results are representative of at least three independent experiments.

Recent evidence shows that EPCs not only participate in tissue repair processe in cardiovascular disorders [16-19], but are also able to mobilize and homing into injured renal medullopapillary region in response to renal injury [20] and injured lungs in elastase-induced lung injury [21]. It would be interesting to investigate whether HBV virus-infected EPCs could be recruited into these injured sites where they were further differentiated to capillary endothelial cells. Since the secondary lung and kidney injury was a frequent complication of acute or chronic MI [22-24], we first thought to check whether the HBV antigens could be detected in the injured lung tissues. Both HBcAg and HBsAg were detected in interstitium of lung (Fig. 7C and 7D) after 2 weeks transplantation with HBV-treated EPCs in MI mice. As expected, no HBV virus antigens were detected in lung tissues when MI mice transplanted with HBV-positive serum alone (containing 2.5 × 10^6 ^copies of HBV DNA) (Fig. 7A and 7B). We then further examined the HBV antigen expression in the injured kidney tissues. Similarly, HBV antigens were detected in renal glomerular vascular endothelium in MI mice with (Fig. 7G and 7H) or without unilateral renal artery clamping (data not shown). No HBV virus antigens were detected in kidney when mice transplanted with HBV-positive serum alone (Fig. 7C and 7D). These data strongly suggested that EPCs may play a critical role in mediating trans-infection of HBV into extrohepatic tissues via the process of EPCs recruitment.

## Discussion

HBV proteins and nucleic acids detected in extrahepatic tissues are tropism in endothelial tissues including endothelium, epithelium, and macrophages [25,26]. Several studies have demonstrated that HBV DNA or HBV antigen was detected in PBMCs [27] or bone marrow cells [11,28,29], suggesting that these cells could be infected with HBV, while others have demonstrated that the HBV is not replicated in PBMCs [13]. It is clear that the evidence for replication of the HBV at extrahepatic sites is controversial or incomplete, and these sites are not usually considered in discussions of viral reproduction and pathogenesis. In this study, we demonstrated in the first time that EPC is a predominant target for HBV infection in vitro. The virus particles detected in this study have the diameters ranged from 50 nm to 200 nm, and most of which are about 80 nm. Similarly, several previous studies have reported 80 nm HBV virus particles in liver biopsy and 70 nm HBV virus particles in kidney biopsy by electron microscopy [30,31]. In contrast, Kendrey et al. reported a virus particle with 80 nm diameter that might be another virus infection [30]. Significantly, the number of viral particles and copies of HBV DNA detected in the virus-treated EPCs were increased in 7 days and were rapidly decreased afterwards in a time-dependent manner (Fig. 4A and 4C). Our results are consistent with another study on hepatitis B antigen in bone marrow cells by Chai et al. [28]. The reason for short life of viral particles and HBV DNA in EPCs is not clear. Several previous studies have reported that HBV replicates in ER and vesicle [32,33]. In our study, EPCs were shown to be short of rough endoplasmic reticulum (RER) and vesicles, which may argue that HBV was unable to replicate in EPCs. Consistently, the negative results for HBV cccDNA detection in the virus-exposed cells further support EPCs were the targets preferable for HBV uptake rather than viral replication.

Recently, Murohara et al. [34] found that cord blood derived EPC transplantation quantitatively augmented neovascularization and blood flow in the ischemic hind limb. Ricousse-Roussanne et al. [35] demonstrated progenitors of both endothelial cells and smooth muscle cells were present in cord blood and that they could differentiate into matured endothelial cells and smooth muscle cells. In our study, EPCs derived from cord blood are characterized by the expression of the early hematopoietic stem cell markers CD34, CD133 and VEGFR-2 (KDR), and can differentiate into cells that display a classical endothelial cell morphology and characteristics, such as the expression of von Willebrand factor and CD31, and the capacity to uptake acetylated low-density lipoprotein. It is worth noting that the angiogenic property of EPCs was also not affected by in vitro HBV infection, indicating that the potential role of EPCs in neovascularization may not be impaired by virus uptake.

EPC transplantation has been shown to induce new vessel formation in ischemic myocardium [36-38] and to accelerate re-endothelialization of injured vessels and prosthetic vascular grafts in humans and in various animal models [39,40]. Our data from transplantation of HBV-treated EPCs in MI-mouse models and in the acute renal ischemia mouse models revealed that EPCs together with HBV were able to incorporate not only into injured intramyocardial capillary and interstitial tissues in the peri-infarct areas, but also into lung and kidney tissues. Our findings support the hypothesis that HBV incorporation into the vascular endothelium is due presumably to the fact that EPCs is capable of uptaking and transporting the virus into the damaged tissues via the process of homing and transdifferentiation.

The pathogenesis of the HBV-related extrahepatic disorders is poorly understood, several mechanisms have been proposed [1,2]. These include the deposition of circulating hepatitis B antigen-antibody complexes in extrahepatic tissues; the local induction of immune complex formation in extrahepatic tissues; viral induction of host autoantibodies reactive with extrahepatic tissues; and extrahepatic viral replication. It is known that the timing of onset of most extrahepatic disease coincides with immune complex formation, which suggests a major role for immune complexes in disease formation with immune and viral consequences in the extrahepatic organs. Theoretically, it would be also possible that the extrahepatic tissue injury caused by immune complex formation at the time of acute (or chronic) infection may trigger the activation of tissue repairing process, which leads to HBV-infected EPCs and/or other bone marrow-derived stem cells recruited into the injured tissues. However, this must be tested experimentally using acute (or chronic) hepatitis animal models. An alternative mechanism by which both immune complexes and HBV replication within the endothelium combine to promote extrahepatic disease has been recently proposed [12]. However, how the immune complexes-mediated tissue injured occurs, and the mechanism by which allows entry of HBV into extrahepatic tissues has not been established. Our study clearly demonstrates that EPCs are capable of uptaking active HBV virus that facilitates virus incorporation into injured endothelial tissues. These results are supportive of a novel role for EPCs in mediating HBV-related extrahepatic disorders, which may account for most clinic transient or asymptomatic HBV-related extrahepatic manifestations such as vasculitis, myocarditis, glomerulonephritis, and polymyositis. However, how re-colonization of HBV by EPC uptake results in the extrahepatic disorders still remains to be elucidated. Since our data do not support viral replication in EPCs, it is difficult to envision the extrahepatic manifestations by HBV resulting from direct uptake of virus. Nevertheless, it should be pointed out that our results can not rule out the possibility of viral replication in the regenerated endothelial cells that differentiated from HBV-infected EPCs. It is possible that virus uptake in EPCs allows virus entry into the injured tissues, which would in turn mediate a secondary infection and/or a local immune complex formation in the extrahepatic tissues via the activation of tissue repairing machinery. Given the complexity of extrahepatic disorders, further studies are required to address (i) whether re-colonization or incorporation of HBV in EPCs and in the extrahepatic endothelial cells could be reactivated to infect the neighboring cells and tissues; (ii) whether and how the incorporation of virus into the extrahepatic endothelium has pathological consequence on the injured organs.

## Conclusion

In conclusion, our results demonstrate that HBV can be effectively uptaken into EPCs in vitro. HBV uptake had no inhibitory effects on EPCs angiogenic potential. Transplantation of EPCs into MI and acute renal ischemia mouse models revealed that EPCs can serve as a virus reservoir harboring and transporting HBV to the injured endothelial tissues. The above data support to a novel role of EPCs in mediating virus associated extrahepatic diseases including virus relevant tumor diseases.

## Methods

### Chemicals and antibodies

Chemicals and antibodies were as follows: 1,1'-dioctadecyl-3,3,3',3'-tetramethylindocarbocyanine-labeled acetylated LDL (DiI-acLDL, Molecular Probe); fetal calf serum (Hyclone); VEGF, and FITC-conjugated goat anti-mouse IgG antibody (Cytolab); Medium 199, bovine brain extract, and goat polyclonal anti-von Willebrand factor (vWF) (Sigma); FITC-conjugated mouse monoclonal anti-human CD34 (Immunotech); mouse monoclonal anti-VEGF receptor 2 (KDR) and FITC-conjugated goat polyclonal anti-HBsAg (Ad/Ay) (abcam); R-Phycoerythrin (PE)-conjugated mouse monoclonal anti-human CD133 (Miltenyi Biotec, Bergisch Gladbach, Germany); mouse monoclonal anti-HBcAg (ayw, USBio); goat polyclonal anti-CD31 antibody (Santa Cruz Biotechnology); mouse monoclonal anti-hepatitis B virus surface antigen (HBsAg) (Dako, Denmark), rabbit polyclonal anti-hepatitis B core antigen (HBcAg) (Maoxin, China); anti-human leukocyte antigen HLA-ABC (Chemicon); goat anti-rabbit IgG Texas red conjugate (Calbiochem); horseradish peroxidase-conjugated anti-goat IgG (Sigma); epoxy resin (TAAB 812; Emmer Green, Reading, England); DAB staining kits (Wuhan BoShiDe Biological Engineering Ltd.); cyclosporin A liquid (Fujian China Kerui Medicine Co. Ltd.); OCT medium (Sakura, USA). All other chemicals were of the highest grade commercially available.

### Preparation of virus

HBV-positive serum samples (≥ 1 × 10^6 ^copies of HBV DNA/mL) were obtained from the patients with chronic HBV infection. Sera were filtered through 0.22-um pore-sized filters and stored at -20°C. As a normal control, serum specimens were obtained from healthy human volunteers with no history of HBV exposure and lacked serologic evidence of previous or current HBV infection.

### Isolation and cultivation of human EPCs

Human cord blood was collected from umbilical vein from healthy newborn donors by a protocol approved by the ethical committee of the Medical Faculty of Nanjing medical University. Mononuclear cells (MNCs) were isolated by density-gradient centrifugation using 1.077 g/mL Histopaque solution. Human umbilical vein endothelial cells (HUVECs) were isolated according to the method of Jaffe et al. [41]. Both MNCs and HUVECs with a density of 2 × 10^6 ^cells/cm^2 ^were plated on fibronectin-coated dishes (2 μg/cm^2^, Chemicon) and cultured in Medium 199 supplemented with 20% fetal calf serum, 12 μg/mL of bovine brain extract, 10 ng/mL of VEGF, penicillin (100 U/mL), and streptomycin (100 μg/mL).

### Infection of EPCs with HBV

For cell infection, freshly isolated MNCs were cultured in Medium 199 containing either 10% of HBV-positive (virus-treated) or normal control serum (mock-treated) for the first 4 days. After 4 days culture, free virus and nonadherent cells were removed and fresh media were replaced. The cells were further cultured for additional 3 days. The numbers of spindle-shaped and attached cells and cell clusters were counted under microscope at day 4 and day 7 of culture. Five randomly selected microscopic fields were evaluated and mean numbers of attached cells and cell clusters were calculated.

### HBV DNA extraction and quantification

Approximately 10^6 ^EPCs and HUVECs were treated with proteinase K in the presence of sodium dodecyl sulfate, and HBV DNA was then purified by DNA extraction kit (Tiangen, Beijing) according to the manufacturer's protocol. HBV DNA contents were determined by quantitative real-time polymerase chain reaction (qRT-PCR) using HBV fluorescence quantitative PCR diagnostic kit (Daangene, China). Primers used for HBV DNA detection were: 5'-ATCCTGCTGCTATGCCTCATCTT-3' (forward), and 5'-ACAGTGGGGAAAGCCCTACGAA-3' (reverse). The probe used was 5'-TGGCTAGTTTACTAGTGCCATTTTG-3'. HBV covalently closed circular DNA (cccDNA) quantification was performed as described previously [42]. The primers used for HBV cccDNA quantification were: 5'-ACTCTTGGACTCBCAGCAATG-3' (forward), and 5'-CTTTATACGGGTCAATGTCCA-3' (reverse). The probe used was 5'-CTTTTTCACCTCTGCCTAATCAT CTCWTGTTCA-3'. A plasmid containing Chinese HBV genome (pHBV-adr) (a gift from Professor Yuan Wang) was used as positive control.

### Histology, immunohistochemistry, and immunofluorescence

Isolated mouse tissues were fixed in 10% formaldehyde, embedded with paraffin and cut into 4 μm thick sections. The tissue sections were then rinsed with PBS for 3 times, blocked with 5% horse serum in PBS at room temperature for 30 min. For HBV antigens detection, the sections were incubated with mouse monoclonal anti-HBsAg and rabbit polyclonal anti-HBcAg, and were detected by DAB staining kit according to the manufacturer's protocol. For immunofluorescent double-staining, the sections were incubated with rabbit polyclonal anti-HBcAg antibody at room temperature for 1 hr, followed by anti-HLA-ABC for a further 1 h. Sections were then extensively washed and incubated with Texas Red-conjugated anti-rabbit IgG and FITC-conjugated anti-mouse IgG (both 1:100 dilution) at room temperature for 30 min. After extensive washing, the sections were mounted and visualized using fluorescent microscope (Nikon, Japan).

For detection of endothelial cell surface markers, the cells were plated on 6-well chamber slides for 7 days, and then washed and fixed with acetone at 4°C for 10 min. The cells were blocked with 5% horde serum for 30 min and incubated overnight at 4°C in 1:100 dilutions of goat polyclonal anti-vWF and CD31 antibodies, followed by incubation with horseradish peroxidase-conjugated anti-goat IgG for additional 30 min. The slides were visualized with DAB-based color development. For detection of HBV antigens, the cells were incubated with mouse monoclonal anti-HBsAg and rabbit polyclonal anti-HBcAg, and were detected by DAB staining kit according to the manufacturer's protocol.

### Fluorescent microscopy and electron microscopy

Fluorescent chemical detection of EPCs was performed on attached cells after 7 days in culture. Direct fluorescent staining was used to detect DiI-acLDL of the cells. Briefly, adherent cells were washed with fresh medium and incubated with 10 μg/mL of DiI-acLDL at 37°C for 4 hrs. Cells were then fixed in 2% paraformaldehyde for 10 min and the number of EPCs was examined under fluorescent microscope (Nikon 120). For electron microscopy analysis, cells were scraped and first fixed in 2.5% glutaraldehyde for 3 hrs, followed by 1% osmium tetroxide in 100 mM phosphate buffer for additional 30 min. After dehydration, tissues were embedded in epoxy resin. Ultrathin sections were stained with uranyl acetate and lead citrate, and were visualized under transmission electron microscope (JEOL 1010, Japan).

### Fluorescence activated cell sorting (FACS) analysis

For EPC characterization, cells were detached with 1 mM EDTA followed by repeated gentle flushing through a pipette tip. Cells (2 × 10^5^) were incubated for 30 min at 4°C with FITC-conjugated mouse monoclonal anti-human CD34, mouse monoclonal anti-VEGF receptor 2 (KDR), and R-Phycoerythrin (PE)-conjugated mouse monoclonal anti-human CD133 antibodies. After extensive washing, the cells were incubated with FITC-conjugated goat anti-mouse IgG antibody for additional 30 min. FITC-conjugated isotype-identical IgG (Caltag Lab) served as negative controls. Flow cytometric analyses were performed by FACScan and Cell Quest software (Becton, Dickinson). Each analysis included at least 50 events.

For viral proteins detection, cells (1 × 10^6^) were fixed and permeabilized as described previously [43], followed by incubation with FITC-conjugated goat polyclonal anti-HBsAg and mouse anti-HBcAg at room temperature for 20 min. Cells were then washed with PBS and incubated with FITC-conjugated goat anti-mouse IgG antibody at room temperature for additional 20 min. After washing, the cells were resuspended in 0.3 ml of PBS and analyzed by FACS.

### In vitro tubule formation assay

In vitro tubule formation assay was performed with In Vitro Angiogenesis Assay Kit (Chemicon) according to the manufacturer's instructions. Briefly, ECMatrix™ solution was thawed on ice overnight then mixed with 10 × ECMatrix™ diluents and placed in a 96-well tissue culture plate at 37°C for 1 h to allow the matrix solution to solidify. Cells were plated (10,000 cells/well) on top of the solidified matrix solution, and were grown with HBV positive serum or normal control as described above for 3 days. Tubule formation was determined under an inverted light microscope at 200× magnification. Tubule formation was defined as a structure exhibiting a length four times longer than its width. Five independent fields were assessed and the average numbers of tubule branch points were determined.

### Induction of myocardial infarction (MI) and acute renal ischemia

Male Sprague & Dawley mice weighing 150 to 250 g were used (Nanjing Medical University Laboratories). Mice were anesthetized with Ketamine 100 mg/kg and were ventilated with 100% oxygen using a rodent ventilator (Nanjing Medical Apparatus, Inc). The chest was opened, a 6-0 silk suture was passed with a tapered needle under the left anterior descending coronary artery 1 to 2 mm from the tip of the left atrium, and the two ends of the suture were tied to induce MI. In some experiments, mice were subjected to unilateral renal artery clamping for 25 min after induction of MI. Sham-operated mice received the same procedure except left anterior descending ligation and unilateral renal artery clamping.

### Transplantation of EPCs in mice

Two and a half million human EPCs that cultured in Medium 199 containing 10% of HBV-positive serum for 4 days and changed Medium 199 containing 10% of HBV-positive serum for another 3 days were injected through the testicle vein of 15 mice 4 hrs after MI induction. Cyclosporin A was administrated 24 hrs before the operation to suppress immune rejection. All experimental animals were given cyclosporin A at dosage of 10 mg/kg/day during the experiments. The hearts were harvested at 1 week (n = 3), 2 weeks (n = 3), 3 weeks (n = 3) and 4 weeks (n = 3) after cell transplantation. The lungs and kidneys were harvested at 2 weeks (n = 3). Another group of 6 mice with MI surgical manipulation (n = 3) or sham surgery (n = 3) was injected with the same number of non viral infected EPCs (normal intervened) through the testicle vein and killed at 2 weeks or 4 weeks after transplantation.

### Tissue harvesting

Hearts, lungs, and kidneys were removed. Half of each was immerged in 10% paraformaldehyde and embedded in paraffin. Another half was embedded in OCT medium followed by snap frozen in liquid nitrogen, and stored at -80°C before analysis. Mouse bloods were also collected and stored at -20°C for detecting HBV DNA by PCR.

### Statistical analysis

All values are presented as mean standard error. All data were subjected to unpaired Student's t-test for comparison between two means. P values < 0.05 were considered to be statistically significant.

## Competing interests

The author(s) declare that they have no competing interests.

## Authors' contributions

RQ: Designed and carried out most of the studies, analyzed sequences and interpreted results. Participated in the preparation of the manuscript.

HJ: Responsible for the design of the study and for acquiring financial support.

Supervision of the experimental work. Participated in the drafting of the manuscript.

SE: supervised molecular techniques and participated in the designed of the study and in the analysis of results.

LJ, Zl and CK: Participated in the designed of the study and in the analyses and interpretation of the results.
